# Elevating the impact of conservation physiology by building a community devoted to excellence, transparency, ethics, integrity and mutual respect

**DOI:** 10.1093/conphys/coac015

**Published:** 2022-04-01

**Authors:** Steven J Cooke, Kevin R Hultine, Jodie L Rummer, Nann A Fangue, Frank Seebacher, Erika J Eliason, Heath A MacMillan, Andrea Fuller, Craig E Franklin

**Affiliations:** Fish Ecology and Conservation Physiology Laboratory, Department of Biology and Institute of Environmental and Interdisciplinary Science, Carleton University, 1125 Colonel By Dr., Ottawa, Ontario, K1S 5B6, Canada; Department of Research, Conservation and Collections, Desert Botanical Garden, 1201 N Galvin Parkway, Phoenix, AZ 85008, USA; College of Science and Engineering and ARC Centre of Excellence for Coral Reef Studies, James Cook University, Townsville, Queensland, 4810, Australia; Department of Wildlife, Fish, and Conservation Biology, University of California Davis, Davis, CA 95616, USA; School of Life and Environmental Sciences, The University of Sydney, NSW, 2016, Australia; Department of Ecology, Evolution and Marine Biology, University of California, Santa Barbara, Santa Barbara, CA 93106, USA; Department of Biology and Institute of Biochemistry, Carleton University, 1125 Colonel By Dr., Ottawa, Ontario, K1S 5B6, Canada; Brain Function Research Group, School of Physiology, University of the Witwatersrand, Johannesburg, 2000, South Africa; School of Biological Sciences, The University of Queensland, Brisbane, Queensland , 4072, Australia

## On conservation physiology

Ten years ago, the journal *Conservation Physiology* was launched jointly by the Society for Experimental Biology and Oxford University Press. Much has been accomplished since 2012 including publishing over 600 papers in the journal and helping to build a sense of place for aspiring and practicing conservation physiologists (Cooke *et al.*, 2020). Yet, more work is needed to further elevate the impact of conservation physiology as a discipline and community. Here, we summarize what is needed to build and strengthen a community devoted to not only excellence, transparency, ethics, integrity and mutual respect, but also courage to tackle some of the overarching challenges humanity faces. As active voices in the conservation physiology community we hope that this paper will help shape the future of our discipline while also guiding the activities and priorities of the journal and editorial team.

Since the term ‘conservation physiology’ was coined by Wikelski and Cooke (2006) it has emerged as an essential component of conservation science and practice. Conservation physiology is about the use of physiological tools, knowledge and concepts to understand and solve conservation problems across diverse taxa (Cooke *et al.*, 2013). It is regarded as being particularly effective at understanding mechanisms, generating cause–effect relationships (e.g. threat X does Y to organism Z), creating predictive tools and testing conservation interventions (Cooke and O’Connor, 2010). Issues relevant to conservation physiology range from very local, focused on recovery of an imperilled population (Birnie-Gauvin *et al.*, 2017), to global-scale issues such as tackling the UN Sustainable Development Goals (Cooke *et al.*, 2020) and the climate crisis (Madliger *et al.*, 2021c). The discipline is now supported by a conceptual framework (Coristine *et al.*, 2014), a journal (https://academic.oup.com/conphys) and a reference book (Madliger *et al.* 2021a). There is also a growing community of researchers who engage in conservation physiology and even define themselves as conservation physiologists (Madliger *et al.*, 2021b). Moreover, in conservation physiology there are success stories that demonstrate the potential of conservation physiology (Madliger *et al.*, 2016).

## Growing pains

As with any nascent discipline, especially one that demands generating actionable knowledge that can be used by decision-makers and practitioners, there are also a number of challenges (Cooke and O’Connor, 2010; Madliger *et al.*, 2021b). Indeed, there is a well-defined knowledge–action gap in conservation and numerous barriers influence the extent to which science is used by conservation practitioners and decision-makers (Cook *et al.*, 2013). Madliger *et al.* (2021b) surveyed those working in conservation physiology and revealed that key barriers include the relative ‘youngness’ of the discipline and issues with translating science in usable forms. Issues such as trust, relevance, saliency and institutional norms and inertia are also well understood as barriers to knowledge use in conservation (Rose, 2015). Because conservation physiology is a relatively new field the evidence base remains small. A robust evidence base is relevant in that decision-makers increasingly rely on a body of evidence (or should!), ideally presented in the form of a synthesis such as a systematic review with meta-analysis, to inform decisions (Thomas-Walters *et al.*, 2021). Growing the evidence base with high-quality empirical research is a precursor to evidence synthesis and evidence-informed decision-making. In contemporary evidence synthesis, not all evidence is considered equal (Haddaway *et al.*, 2017). Appraisal of study quality is important, and studies with experimental design deficiencies (e.g. low sample size, lack of relevant controls) are excluded or down-weighted. Cooke *et al.* (2017) have previously called for the conservation physiology community to up their game in an effort to increase the likelihood that conservation physiology studies can be used in evidence synthesis and be embraced by decision-makers.

One of the problems plaguing science today is the so-called ‘replication crisis’ (Ioannidis, 2005; Loken and Gelman, 2017), which has led to questions about the reliability of the evidence bases needed to support all forms of decision-making [e.g. from medicine (Lipscomb, 2021) to psychology (Maxwell *et al.*, 2015) to environmental issues and conservation (Bennett *et al.*, 2017a)]. In simple terms, the replication crisis (also known as the reproducibility crisis; Fanelli, 2018) is a failure of scientists to successfully replicate studies, which can lead to significant uncertainty and distrust among the public and decision-makers (Hendriks *et al.*, 2020). The basis for the replication crisis is complex and can include measurement error (Loken and Gelman, 2017), publication bias whereby only studies with significant findings are published while those without stay in the proverbial file drawer (Scargle, 2000) and, in rare cases, scientific misconduct. Measurement error can be addressed through improvements in scientific methods and analyses. The file drawer effect can be addressed by placing equal weight on non-significant findings assuming that studies are well designed and have acceptable statistical power (Reed, 2018; see below for details on what *Conservation Physiology* is doing to address this). Another phenomenon that has been documented in several fields, including ecology, is the ‘decline effect’ whereby early studies on a topic document extreme effect sizes with highly significant findings (Jennions and Møller, 2002). Over time, those effects become less apparent, although the file drawer effect can mask the decline effect (Schooler, 2011). A recent analysis of ocean acidification effects on fish (which is salient to conservation physiology) suggests evidence of a major decline effect where large effects in early studies have all but disappeared in subsequent studies, over a decade (Clements *et al.*, 2022). Explanations for this pattern are varied and could include early studies being focused on fish in coral reef habitats. What is important here is that evidence synthesis (including meta-analyses) has the potential to account for various factors that could influence findings and ensure that all high-quality evidence is considered rather than focusing solely on studies with major effect sizes that are often published in top tier journals (Sharpe and Poets, 2020).

## Embracing integrity and excellence in research

Expectations to publish have never been greater than for today’s early- and mid-career scientists. As a consequence of heightened expectations to rapidly build bibliometric profiles, the fundamental approach to data collection and interpretation have evolved so that the way in which the basic tenets of the scientific method are practiced is ‘watered down’ (Kerr, 1998). We advocate for a scientific approach that emphasizes publication quality and influence (Donaldson and Cooke, 2014) over quantity. An increased emphasis on quality could lead to more long-term studies with larger sample sizes that produce more robust statistical power. Longer-term studies with larger datasets could also reduce the pressure to avoid publishing negative or non-significant results, which in turn will reduce publication biases, as long as journal outlets increase their willingness to publish non-significant results. Likewise, long-term studies with richer datasets could reduce time-lag bias (Nakagawa *et al.*, 2021) that can arise in cases where statistically significant effects are published more rapidly than are non-significant or smaller effects, leading to a decline over time in the magnitude of the overall effect (Koricheva and Kulinskaya, 2019). This is not to say that high-quality research can only be derived from ‘slow science’ or long-term studies—what matters most is that the evidence generated is robust (Roche *et al.*, 2019). A reduced emphasis on publication quantity could have other benefits, such as helping to level the playing field where many early-career scientists are disadvantaged relative to their peers in cases where English is a second language or when demographic groups are required to absorb an oversize burden in the face of hardships (e.g. early-career female professionals dropping out of the workforce in higher numbers than their male counterparts as a consequence of COVID-19; Squazzoni *et al.*, 2021). The reasons for hyper-productivity are varied and some individuals and laboratory groups will produce more science than others (which is fine…run your own race), but at the end of the day quality and impact should always be prioritized over quantity. Coupled with an enhanced emphasis on data transparency, emphasizing publication quality over quantity will greatly improve access by practitioners and trust between practitioners and scientists, which in turn could facilitate better scientific communication with the public at large.

A high degree of ethical behaviour and integrity is required from all scientists. However, in branches of biology (such as conservation physiology) that aim to contribute to the evidence bases that ultimately guide decisions by governments and policy-makers, the consequences of lapses in integrity are potentially more far reaching than in ‘blue sky’ science that is further removed from translational outcomes. Integrity and behaviour are individual responsibilities (Davies, 2019). There is increased pressure on the individual then to scrutinize their own behaviour and scientific approaches when research results can influence conservation outcomes directly. Nonetheless, institutions can contribute to supporting ethical behaviour (Zwart *et al.*, 2019): employee performance reviews should value quality rather than focussing on quantity, granting bodies should adjust expectations of research output in view of the potential impact of the research and journals and their editors can guide authors and be more open to publishing non-significant findings.

During the publication process, editors can interact directly with authors to develop manuscripts even if these are ultimately published elsewhere. A journal such as *Conservation Physiology* has the potential to increase integrity and reproducibility of results by setting high standards in experimental design and statistical power of potential contributions and by mandating high levels of transparency. When working on rare or imperilled species or in challenging environments (e.g. the deep sea, polar regions) there is also a trade-off in that sample sizes may be constrained (Bissonette, 1999). At *Conservation Physiology* we are aware of this conundrum and try to balance the need for robust experimental designs with the inherent challenges of working on organisms for which sample sizes will never be large. At the end of the day, the value of information is considered in all decisions at *Conservation Physiology*, but we always ensure that limitations and caveats are utterly clear to readers. Individual integrity has a communal dimension (Mitcham, 2003). By supporting the individual, the community maximizes ethical behaviour. Obviously, the more cohesive the community, the better able it will be in influencing individual behaviour (Steneck, 2006). Hence, lapses in ethical behaviour by individuals may reflect dysfunction in the community to a certain extent (Mitcham, 2003; Steneck, 2006). In cases of questionable behavioural ethics, it may therefore be more constructive to reflect on our own behaviour as a group and how we can strengthen the integrity within the field rather than just pointing the finger at individuals. Nonetheless, there are some instances when there is a need to rely on formal processes when there are more egregious issues (e.g. accusations of academic misconduct), but ‘innocent until proven guilty’ must be the default. Of course, that means that institutions must be willing to step up and lead such investigations in a transparent, timely and objective way when necessary—something that remains uncommon.

## Encouraging ethical behaviour and building integrity

Sometimes honest mistakes do happen in science. For example, incorrectly transcribed data, an error in code or a typographical error in a data table can alter the outcome of a study. No one is perfect, and hopefully these issues are resolved well before the manuscript is published. However, sometimes a mistake slips through. When these issues are discovered (either by authors or external parties), we encourage the highest standard of ethical behaviour. Scientists should rapidly disclose the discovery of their own mistakes to the journal and request a correction. Similarly, should an error occur during production, a correction should be requested (noting whether it is the fault of the publisher or authors).

Open data and improved transparency make it possible to scrutinize other researchers’ data (Roche *et al*., 2022). *Conservation Physiology* embraces the spirit of the FAIR principles (see https://www.go-fair.org/fair-principles/) whereby data (and code) associated with papers are findable, accessible, interoperable and repeatable. As a journal we are just transitioning from encouraging such actions to a requirement for full data accessibility (including analytical code) as a requirement for publication. At the outset, we advise to give people the benefit of the doubt. First, alert the corresponding author of potential concerns. If those concerns are not adequately addressed by the corresponding author working with the journal, then alert the journal directly.

Finger pointing and argument are as old as science itself (Dellsén and Baghramian, 2021). What has changed now is the impact of access to information and ‘cancel culture’ (Norris, 2021) on the scientific community. Open data and transparency policies make it possible to scrutinize data in a way we never could before, and those who find evidence of wrongdoing sometimes want those who cheat the system to be shamed. From the perspective of the public, those repeatedly found of wrongdoing are wasting taxpayer money by misdirecting science (and by extension policy), and they should be relinquished of the power to do more harm. How do we find the line, as a community, where ultimate punishments are appropriate, and how do we reduce the incentives for cheating in the first place? We do not have an answer but hope that efforts to build a culture of ethical behaviour and principles of integrity will help, as will working together to strive for consensus. Of course, teachers, mentors, institutions (including employers, funders and professional organizations and bodies) and established researchers have an important role to play.

## Ensuring lasting trust by better teaching and mentoring students to be trustworthy

Changes to community perspectives and values rarely happen quickly. Lasting change in values happens generationally (Rokeach, 2008), and those of us with the privilege of training the next generation of scientists also have the responsibility to train them in ethical science and science communication so that they emerge as trusting and trustworthy leaders. The availability of information and the willingness of researchers to call out what they see as questionable findings can have severe negative effects on early-career scientists. In our collective anecdotal experience, trainees are becoming less willing to share their ideas (e.g. at conferences or via social media) for fear of being publicly or privately scrutinized. This issue directly intersects with equity, diversity and inclusion; those trainees and early-career scientists most uncertain about sharing their new (and possibly world-changing) ideas or experiencing the self-doubt known as imposter syndrome (Chrousos and Mentis, 2020) are more likely to identify with groups systematically excluded from science, technology, engineering and mathematics (STEM) careers (e.g. Lee *et al.*, 2020). This pattern should perhaps not come as a surprise, given the level of attention and community reaction associated with high-profile cases of scientists that misrepresented their work. Even if trainees are not those who have acted inappropriately, entire trainee careers can become collateral damage because of co-authorship. For these individuals, their hard-earned undergraduate or graduate-level publications have been scrutinized, their faith in the scientific process tested and their confidence and sense of belonging (one of the most critical factors to graduate retention in STEM; O’Meara *et al.*, 2017) challenged. As a scientific community and as mentors, we have a responsibility to stand by and support these early-career scientists when they are victims of the unethical behaviour of their peers, mentors or collaborators.

Mentors of undergraduate students, graduate students and postdoctoral fellows can and should work harder to encourage key traits and behaviours that can disincentivize unethical behaviour and in-fighting and lead trainees towards valuing truth, collaboration and respectful disagreement. This also includes faculty members holding each other accountable to ensure there is a supportive departmental level culture. While consequences for unethical research behaviour can help to correct wrongs after they are identified, a more sustainable solution is to cultivate scientific virtues in our trainees, departments and institutions (Nakamura and Condren, 2018). Individual research groups have an ever shifting ‘laboratory culture’ that emerges organically from group membership and/or is carefully cultivated by mentors through formal codes of conduct. Mentors can choose to emphasize the importance of key traits (e.g. mutual respect and empathy, transparency, a healthy skepticism for one’s ideas and willingness to be wrong), in both formal and informal group communications. These values (and their importance to career advancement) can be emphasized in recruitment ads and web content, formal group policies, welcome packages, meeting topics or other forms of communication. Ideally, these group values should be developed in direct collaboration with trainees through reading and reflection on the topic and undergo regular review and revision from the group as a whole, thereby fostering a sense of belonging and agency in trainees. Importantly, emphasizing the value of transparency, ethical behaviour and respect may help to shift attitudes in trainees, but only if mentors themselves behave in a way that clearly aligns with these stated values.

Finally, as a community we also must rally around early-career researchers who have been sideswiped by nefarious behaviour by their advisors or collaborators. Early-career researchers have been disproportionately impacted by these extremely challenging situations. As a scientific community, it is up to us to stand by and support these valued members of our community who have been victims. And it takes more than a tweet of support. These folks (undergraduate students, graduate students and postdoctoral fellows) need someone to step in as their advisor, offer postdoctoral positions, provide collaboration opportunities and to not judge them as tainted for having unknowingly been mentored by someone who did not have their best interests in mind.

## In search of respect and kindness

One positive outcome of the COVID-19 pandemic, which has been associated with a rise in mental health burden, is an increased awareness of the need to show compassion, cultivate social belonging and create supportive networks (Slavich *et al.*, 2021). In many ways, the stressors and consequences of the pandemic mirror those in science, where the current competitive academic system can discourage cooperation and place scientists under tremendous pressure to publish. A high prevalence of anxiety and depression (Evans *et al.*, 2018) and imposter syndrome may deter many talented researchers from remaining in the pipeline, with consequences for diversity in science too.

Our efforts to grow the conservation physiology community are likely to succeed best when scientists have the freedom to pursue their own research interests while having a sense of belonging to the research community. Of course, many scientists already contribute to collegiality and mutual respect in such a community through engaging in selfless activities such as reviewing, writing testimonials, freely sharing their resources and knowledge and gaining genuine pleasure in seeing and sharing the successes of other researchers. Continuing education and training on topics such as microaggressions, inclusive excellence and implicit bias is also needed. Kindness in science is increasingly recognized as an important aspect of how we do science (Jost *et al.*, 2021) whether it be our interactions with non-science partners (e.g. stakeholders, rights holders, decision-makers), collaborators or trainees. Unfortunately, however, the critical nature of the scientific method can often result in harsh commentary and conflict.

Peer review is one place where kindness is essential yet is often in short supply (Clements, 2020). Comments that may be perceived as relatively innocuous by the reviewer, such as those related to English language ability (Romero-Olivares, 2019), can have profound impacts, particularly for those early in their career. As reviewers, we need to recognize biases that may affect our perceptions as well as make greater efforts to provide positive and constructive feedback. Peer review should not be about crushing souls but rather about elevating our collective science and ensuring that it is shared in a manner that is as clear and cogent as possible. No matter how frustrated referees are, it is important for them to remember that there is another human at the receiving end of those comments (Yoon *et al.*, 2021). Scientists are learners and no matter how senior or eminent—or junior and novice—there are opportunities to learn from peer review, but that requires sharing criticisms in a kind way. We can also be more respectful of the peer review process and kinder to editors and authors by responding timeously to requests to review and providing alternative reviewers when we cannot assist.

As a journal we can institute policies and practices yet at the end of the day, kindness is central to our nascent discipline and is hard to regulate. At *Conservation Physiology* we certainly desire our referees to assess work through a critical lens but it also needs to be done in a thoughtful way (Fontúrbel and Vizentin-Bugoni, 2020). The same goes for interactions at conferences or even sparring through commentaries on papers. The key is to keep communication civilized and focused on trying to elevate the science and our community. Public shaming (often by social media) has become far too common in the sciences (see Thérèse and Martin, 2010). Why cannot we instead create a culture where intelligent and kind discourse is part of the scientific process?

Beyond the need for kindness in our community is the need for ensuring that we also include kindness in our approach to dealing with massive challenges related to planetary health such as the biodiversity crisis and climate change (Logan *et al.*, 2020). Although conservation science is often focused on wildlife (including all non-human life forms), it is the intersection of people and wildlife (or the environment more broadly) that dictates what is possible in terms of policies and practices (Bennett *et al.*, 2017b).

## Rethinking impact

Universities, institutions and organizations where we work are rapidly changing the way success is measured in science and are including more wide-reaching, inclusive and useful metrics and key performance indicators into performance reviews. Nonetheless, it is also up to us to advocate for the reach of our science and impact of our findings beyond the traditional citation race and impact factor chase. We, as a community, need to emphasize and place more value on the reach of our work and the accessibility of our findings, and we need to integrate the tools for doing this into our own practices, repertoire and even in course materials, lectures and presentations. After all, who is science for? Is it for other scientists? Or, is science for everyone? That said, we should be shifting from emphasizing traditional indicators of scientific impact, such as

- total number of publications,
- total number of citations,
- impact factor of journals,
- h-Index or i10-index of productivity and impact and
- citations per paper.

That shift should be towards, as one example, alternative metrics that account for the reach of our work, which may be particularly relevant in conservation, where informing the public about mitigating environmental issues can be crucial (Bornmann, 2014). Simply put, alternative metrics (or ‘altmetrics’) can trace the somewhat invisible threads that link scientific publications to a not-necessarily-scientific audience (Ravenscroft *et al.*, 2017). These communication forms can come through but are not limited to the following:

- continuous knowledge exchange as is common with co-production research methods (Norström *et al.*, 2020),
- social media (see Bik and Goldstein, 2013 for getting started),
- blogs and websites,
- newspaper articles,
- television interviews and
- radio.

Altmetrics are essentially science’s equivalent of the business world’s web analytics, incorporating online mention of your work. Data to support altmetrics are harvested from the above sources as well as online reference managers, which are then scored based on your online activity. You can report an ‘altmetric score’, therefore, for every scientific contribution you make, and this score indicates that you have opened pathways to communicating your work. Bear in mind that the altmetric score (or any other similar metrics, e.g. SciVerse, Plum Analytics, ImpactStory, etc.) does not gauge the quality of your science or the relevance to policy-makers, just public attention (positive or negative) and the online reach of your work. Yet, steps have been made to incorporate such scores into evaluation mechanisms (e.g. grant panels, tenure and promotion committees, award panels), how we value research outputs (Piwowar, 2013) and even the U.S. National Science Foundation has moved to ‘value all research products’, which includes altmetrics. This is not to say that your Twitter following, for example, should be part of your promotion package. Yet, incidentally, it has been noted that highly tweeted studies were 11 times more likely to be highly cited (Eysenbach, 2011), and in ecology and conservation fields, social media engagement was linked to higher traditional scholarly metrics (Lamb *et al.*, 2018). Nevertheless, these altmetrics are only a first step, in some cases, to making our findings accessible and should become part of our success barometer.

Limitations in disseminating scientific findings and making them accessible have already been experienced by all of us since early 2020 with the global pandemic (e.g. conferences and field work cancelled, moving to online learning and communication), but it is important to note that limitations and accessibility issues with scientific findings have perhaps always been experienced by many communities and demographics worldwide. Because *Conservation Physiology* is an open-access model journal, we do not have *all* of the limitations in disseminating our findings as other scientists do when publishing in other journals. In essence, anyone in the world with an internet connection can access *Conservation Physiology* articles. Yet, the language and format may still be an accessibility barrier. Are those working outside of academia who make management and conservation decisions necessarily going to sift through the traditional format of a journal article and the jargon used to find the pieces of relevant information to make evidence-based decisions that inform policy? Maybe not. Do secondary school teachers have the time or bandwidth to do the same but to find new ideas, developments and conservation issues to communicate to the next generation in their classrooms? Maybe not. However, such stakeholders and ‘next users’ of conservation physiology findings can find the relevant messages through more accessible channels, such as those previously mentioned, where avenues for communicating science use more accessible language, visuals and media. Sections like ‘Conservation Physiology in Action’ in *Conservation Physiology*, where 500-word editorial- or journalism-style pieces are written about up-and-coming studies (i.e. whether they have been published in *Conservation Physiology* or another journal) coupled with a captivating illustration are published almost monthly and highlighted on social media. This not only highlights the work and authors and helps to train young scientists to communicate in a non-jargon, accessible way, but it also gives another avenue of accessibility to those that may need the information. Graphical and video abstracts that are now required by many journals do the same. This need for alternative communication strategies applies to all scientific findings, no matter if they are published in an open-access journal or not. Indeed, there is a profound need to advocate for science communication training, the use of alternative avenues for disseminating our work and putting pressure on those deciding if we are successful or not, including our colleagues and peers, to incorporate the reach of our work and uptake of our findings into those success metrics.

Key performance indicators need to shift accordingly as well. While we have advocated for including altmetrics into gauging our performance, other non-traditional metrics need to be considered, including but not limited to the following:

- number of reads/downloads of peer-reviewed papers (assuming not all papers are downloaded by other scientists);
- briefings to government, management, industry and business;
- public outreach programs;
- public talks, lectures and seminars;
- organizing writing retreats, working groups and workshops; and
- professional development, training courses and research training for students, staff, stakeholders and next-users, including laboratory/team meetings and practice talk sessions.

If we, as conservation physiologists, can first commit to putting more emphasis on these accomplishments (e.g. by revamping our CVs, including statements of impact and reach in grant applications, award nominations and tenure/promotion dossiers) with ourselves, and second, highlight those achievements in others (i.e. with at least equal if not more emphasis than a new journal publication) and underscore how important these accomplishments are to the next generation, then we can make steps towards changing the model of success. Changing the model by which we gauge our success and that of our colleagues will help in advocating for equity, inclusion and accessibility with those we work with and where the impact of our work is felt.

## Change at *Conservation Physiology*

The editorial team at *Conservation Physiology* takes the issues raised here seriously. Here are some of the changes that we have made or will be making for 2022.

- A requirement for data associated with accepted manuscripts to be available in an online, open-access repository. Exceptions will be considered but must be strongly justified (e.g. sharing data on the space use of an imperilled species could lead to its exploitation). This takes effect for all papers submitted beginning 1 January 2022. We provide additional detail and resources on the *Conservation Physiology* website to support authors with this transition. Our goal is to eventually ensure that all data (and code) are shared in accordance with the FAIR principles.
- A requirement for data and code to be shared with referees and editors should it be requested as part of the peer review process.
- A commitment to investigate any issues that arise related to published content in the journal and to do so fully, rapidly and objectively. Readers are encouraged to contact the editorial team should they have concerns about the academic integrity of any content. Our team has experience with such investigations and has adopted best practices (see Bolnick, 2021, for a good overview of what we aspire to do) intended to balance the importance of maintaining the integrity of the scientific record with ensuring that all investigations are done in a fair manner. Expressions of editorial concern or retractions will be used where merited. It is our preference that issues are brought directly to our attention rather than relying on PubPeer (as but one example), given the reasons outlined by Bolnick (2020).
- A commitment to creating opportunities for trainees to learn about best practices for research. This will be achieved in partnership with the Society for Experimental Biology, which has a long history of supporting the development of early-career researchers through webinars and training sessions.
- A continued willingness to give equal weight to null effects provided the experimental designs are rigorous. The file drawer effect must be overcome.
- A continued willingness to accept replication studies. Papers that replicate existing studies will not be rejected based on lack of novelty. We have created a new category of manuscripts called ‘Replication Studies’ that is specifically for papers that test whether previously published experiments can be replicated. As part of the process we are particularly interested in submissions where authors of replication studies engage with those whose work they are trying to replicate (assuming they are still active in science). This is intended to enable more meaningful and efficient truth seeking while minimizing conflict.
- A commitment to creating an ethos of kindness and respect for all interactions within our community. This is particularly relevant to the peer review process where our approach will be thoughtful and supportive with a focus on providing guidance to improve content even if material is deemed inappropriate for *Conservation Physiology*. Referees unable to deliver critiques in a respectful and professional manner will be excused from future review duties. We have also added a message to peer reviewers in the reviewer portal reminding them to use a respectful and constructive tone.
- All papers in *Conservation Physiology* will require a statement of author contributions that acknowledges the ways in which individual authors contributed to the work and the elements for which individuals accept responsibility. We adhere to the CRediT approach (Allen *et al.*, 2014) but recognize that contributions may be more diverse and extend beyond traditional views of authorship (Cooke *et al.*, 2021b).

## The future we desire

We wish to end by thanking the members of the conservation physiology community. Because we are small, supporting each other, amplifying our collective voice and working together is particularly important. Realizing the goals of conservation physiology—not just in terms of generating actionable knowledge but in creating a community of practice that furthers the development of the next generation of conservation physiology professionals—will require the collective efforts of all. Challenges remain—from the continuing challenges arising from COVID-19 (Cooke *et al.*, 2021a) to different regional capacity and support (e.g. in the global south) to ensuring that all voices are welcomed and embraced (Cooke *et al.*, 2020). So thank you for your ongoing support of this (YOUR!) journal and our community. As always, our eyes, ears and minds are open to your thoughts on what we can do to help further conservation physiology (the discipline—and the journal) and the careers of those devoted to this discipline. We would love to hear from you!

## Conflict of Interest Statement

Most authors hold an editorial position on the journal but were not involved with handling the paper in an editorial capacity.

## Contribution Statement

All authors contributed to conceptualization, writing and editing.
